# Dusp1 modulates activin/smad2 mediated germ layer specification via FGF signal inhibition in *Xenopus* embryos

**DOI:** 10.1080/19768354.2020.1847732

**Published:** 2020-11-27

**Authors:** Zobia Umair, Santosh Kumar, Khezina Rafiq, Vijay Kumar, Zia Ur Reman, Seung-Hwan Lee, SungChan Kim, Jae-Yong Lee, Unjoo Lee, Jaebong Kim

**Affiliations:** a Department of Biochemistry, Institute of Cell Differentiation and Aging, College of Medicine, Hallym University, Chuncheon, Gangwon-Do, Republic of Korea; b Department of Electrical Engineering, Hallym University, Chuncheon, Gangwon-Do, Republic of Korea

**Keywords:** Dusp1, activin, Smad2, Bmp, Smad1, Fgf, Erk, Jnk, *Xenopus*

## Abstract

Activin, a member of the transforming growth factor (TGF-β) superfamily, induces mesoderm, endoderm and neuro-ectoderm formation in *Xenopus* embryos. Despite several previous studies, the complicated gene regulatory network and genes involved in this induction await more elaboration. We identified expression of various fibroblast growth factor (FGF) genes in activin/*smad2* treated animal cap explants (AC) of *Xenopus* embryos. Activin/*smad2* increased *fgf3*/*8* expression, which was reduced by co-injection of dominant negative activin receptor (DNAR) and dominant negative Fgf receptor (DNFR). Interestingly, activin/*smad2* also increased expression of *dual specificity phosphatase 1* (*dusp1*) which has been known to inhibit Fgf signaling. *Dusp1* overexpression in dorsal marginal zone caused gastrulation defect and decreased Jnk/Erk phosphorylation as well as Smad1 linker region phosphorylation. *Dusp1* decreased neural and organizer gene expression with increasing of endodermal and ventral gene expression in *smad2* treated AC, indicating that *dusp1* modulates germ layer specification. *Dusp1* decreased neural gene expression in *fgf8* treated AC, suggesting that Erk and/or Jnk phosphorylation may be involved in *fgf8* induced neural induction. In addition, *dusp1* decreased the reporter gene activities of activin response element (ARE) and increased it for bmp response element (BRE), indicating that *dusp1* modulates two opposite morphogen signaling of dorsal (activin/Smad2) and ventral (bmp/Smad1) tracks, acting to fine tune the Fgf/Erk pathway.

## Introduction

The transforming growth factor ß (Tgf-ß) superfamily includes a large group of secreted cytokines including various Tgf-ßs, Bmps, activin and nodals, involved in a wide array of biological processes necessary for coordinating various aspects of development and homeostasis in living organisms. Signaling for Tgf-ß is mediated by critical transducer proteins, Smads (Feng and Derynck 2005; Heldin and Moustakas 2012; Massague 2012). Post phosphorylation by activated type-1 receptors at their c-terminus, receptor associated R-Smads form a heterocomplex with co-Smads (Smad4) and translocalize to the nucleus where they interact with a variety of specific cofactors, involving transcription coactivators/corepressors that promote finetuning transcription of a number of gene targets (Luo 2017).

In *Xenopus* embryos, activin as a member of Tgf-ß superfamily, was first identified as a morphogen involved in mesoderm induction (Asashima et al. 1990; Smith et al. 1990). Activin is necessary developmentally as the use of a dominant negative activin receptor interrupts mesoderm formation for *Xenopus* in early development. Activin is a potent patterning agent of early *Xenopus* development and its lack leads to severe defects in anterior and posterior structures as well as axial tissues such as the notochord and muscle (Piepenburg et al. 2004). Although, it has been clear that activin acts as a morphogen in developing *Xenopus* embryos, the complex regulatory network associated with its long range effects (Gurdon et al. 1995; McDowell et al. 1997) and ability to activate diverse genes remains unclear.

Fibroblast growth factors (Fgfs)/Ras/mitogen activated protein kinase (Mapk) and interacting molecules are essential in many aspects of life including for early embryonic development, differentiation, and organogenesis, also in scenarios of tissue injury and cancer. In *Xenopus,* Fgf was first reported as a factor being involved in mesoderm formation (Kimelman and Kirschner 1987; Slack et al. 1990). Subsequent research identified *fgf* as an important agent for maintenance of mesoderm, via a feedback loop involving brachyury, rather than for induction of mesoderm (Isaacs et al. 1994; Schulte-Merker and Smith 1995; Kroll and Amaya 1996). With their indispensable role in mesoderm development, various *fgf*s are also involved in neural patterning. Several *fgf*s including *fgf8* are expressed in early posterior dorsal mesoderm, present in the vicinity of the presumptive neuroectoderm (Christen and Slack 1997). In all, however, the interrelationships between activin/Smad2 and Fgfs have not been described in detail.

Crosstalk between Tgf-ß/Smad and Fgf/Mapk pathways has long been identified for embryonic development and adult tissue homeostasis with their interactions being highly context dependent, such as for tooth development (Xu et al. 2008), autophagy induction by Tgf-ß (Kiyono et al. 2009) and progression of aortic disease (Holm et al. 2011).

For *Xenopus* development, we previously deciphered the critical role of Fgf/Mapk in dorsoventral mesoderm patterning in combination with endogenous activin and Bmp during early *Xenopus* development (Lee et al. 2011). For a better understanding of the relationship between Tgf-ß/Smad and Fgf/Mapk pathways in *Xenopus* embryos, we examined the up and down regulated genes for activin and *smad2* treated AC. Increased expression of several *fgf*s and *dual-specificity phosphatase-1* (*dusp1*)*,* an inhibitor of Fgf signaling, were identified. Dusp1 is one of the map kinase phosphatases (Mkps) encoded by highly inducible genes, rapidly expressed in response to mitogenic and/or stress stimuli. Dusp1 is primarily found in the nucleus, and it selectively dephosphorylates stress-activated Mapk’s Erk, Jnk and p38 (Keyse 2008; Shen et al. 2016). We examined the role of *dusp1* in activin/Smad2 and Fgf8 mediated germ layer specification, and found down regulation of several neural and organizer genes when *dusp1* was co-injected with *smad2* and *fgf8*. *Dusp1* increased the expression of endodermal and ventral genes, indicating that *dusp1* has an important role in activin/*smad2* and f*gf* mediated germ layer specification and possibly via targets of Dusp1, involving modulation of Erk and/or Jnk phosphorylation. We found that *dusp1* decreased reporter gene activity of activin response element (ARE) and increased that for bmp response element (BRE), suggesting that there are two opposite regulatory outputs of *dusp1* for activin/Smad2 and Bmp4/Smad1 activity via its modulatory inputs of Fgf/Erk pathway. These results led us to identify Dusp1 as finetuning agent for activin, Bmp and Fgf activity in *Xenopus* embryos, and required for pathway regulation in a highly context dependent biological system.

## Materials and methods

### Ethics statement

This animal study was conducted in accordance with regulations of the Institutional Animal Care and Use Committee (IACUC) of Hallym University (Hallym 2012-76, 2013-130, 2019-79). All research members attended both the educational and training courses for the appropriate care and use of experimental animals at our institution in order to receive an animal use permit. Adult *X. laevis* were grown in approved containers by authorized personnel for laboratory animal maintenance, and they were maintained at a 12 hr light/dark (LD 12:12 hr) cycle and at 18'C and according to the guidelines of the Institute of Laboratory Animal Resources of Hallym University.

### DNA and RNA preparation

All mRNA used for this study was synthesized by linearizing the target vectors with the appropriate restriction enzymes and transcription using the MEGA script kit according to manufacturer’s instructions (Ambion, Austin, TX). The linearizing enzymes for each vector included Flag-Dusp1: Sp6, Kpn1, Flag-Smad2: Sp6, Acc651, DNFR: Sp6, EcoR1, Fgf8a: Sp6, Not1, and 6Myc-Fgf8b: Sp6, Not1. The in-vitro synthetic mRNAs were quantified by a spectrophotometer at 260/280nm (SpectraMax, Molecular Devices, San Jose, CA).

### Embryo injection and explant culture

The *Xenopus laevis* adults were obtained from the Korean Xenopus Resource Center for Research (Seoul, Korea). The *Xenopus* embryos were obtained by in vitro fertilization after induction of female frogs with 500 units of human chorionic gonadotropin (Sigma, St. Louis, MO). Embryo injection involved RNAs being injected into the animal pole of 1–2 cell stage embryos. Animal caps explants were then dissected from injected embryos at stage 7–8 and incubated to stage 11 and 24 in L-15 medium for the RT–PCR experiments (Table 1).

**Table 1.** Primers used for RT-PCR amplification.

Gene name	Sequences	Annealing temp (°C)	Cycles
Zic3	F5’TCTCAGGATCTGAACACCT3’ R5’CCCTATAAGACAAGGAATAC3’	45	28
FoxD5b	F5’ACTCTATCAGGCACAACCTGTC3’ R5’GGTCTGTAGTAAGGCAGAGAGT3’	50	30
Dusp1	F5’-AGGCCCTTGGAATTACAGCC-3’ R5’-AGGCCCTTGGAATTACAGCC-3’	60	26
Chrd	F5’TTAGAGAGGAGAGCAACTCGGGCAAT3’ R5’GTGCTCCTGTTGCGAAACTCTACAGA3’	57	25
Noggin	F5’ AGTTGCAGATGTGGCTCT3’ R5’ AGTCCAAGAGTCTGAGCA3’	57	27
BMP4	F5’ CATCATGATTCCTGGTAACCGA3’ R5’ CTCCATGCTGATATCGTGCAG3’	57	25
Ventx1.1	F5’CCTTCAGCATGGTTCAACAG3’ R5’CATCCTTCTTCCTTGGCATC3’	57	26
Xbra	F5’-GGATCGTTATCACCTCTG-3’ R5’-GTGTAGTCTGTAGCAGCA-3’	57	25
ODC	F5’GTCAATGATGGAGTGTATGGATC3’ R5’TCCATTCCGCTCTCCTGAGCAC3’	55	25
Gata2	F5’AGGAACTTTCCAGGTGCATGCAGGAG3’ R5’CCGAGGTGCAAATTATTATGTTAC3’	57	24
Mixer	F5’CACCAGCCCAGCACTTAACC3’ R5’CAATGTCACATCAACTGAAG3’	55	28
Sox17β	F5’GTCATGGTAGGAGAGAAC3’ R5’ATCTGTTTAGCCATCACTG3’	56	26
EF1α	F5’ CCTGAATCACCCAGGCCAGATTGTG3’ R5’ GAGGGTACTCTGAGAAGCTCTCCACG3’	57	27
Histone4	F5’CGGGATAACATTCAGGGTATCACT3’ R5’ATCCATGGCGGTAACTGTCTTCCT3’	55	28
Fgf3	F5’GTCATTTGTTTCCAGACTTC3’ R5’TATCAGTAGGTGGTACTTAG3’	55	29
Fgf8	F5’ACCAGCCTTCGTACTTGACA3’ R5’CTGGTGACCGACCAACTAAG3’	56	27
Ncam	F5’ CACAGTTCCACCAAATGC3’ R5’ GGAATCAAGCGGTACAGA3’	58	29
Neurod	F5’ GTGAAATCCCAATAGACACC3’ R5’ TTCCCATATCTAAAGGCAG3’	47	29
Actin	F5’GCTGACAGAATGCAGAAG3’ F5’TTGCTTGGAGGAGTGTGT3’	55	24
Scl	F5’GTGATTGAGCTGCTCAGAAG3’ F5’CTGGAGTCAATGATGCTCTG3’	50	26
Edd	F5’CTCGCTCTGGACAAAACTC3’ R5’GAGCTTCTTGATGGGAATG3’	57	25
Globin	F5’CATGGCTCTGCTGATCTGCCAACCAC3’ R5’CCCAGGCTGGTGAGCTGCCCTTGCTG3’	57	26
BF1	F5’ACAGCTCAGTCCTGACTCAA3’ R5’AGTCCTGTAGTGAAGCTTGG3’	65	30
Otx2	F5’ GGATGGATTTGTTGCACCAGTC3’ R5’ CACTCTCCCAGCTCACTTCTC3’	57	27
Krox20	F5’ AACCGCCCCAGTAAGACC3’ R5’ GTGTCAGCCTGTCCTGTTAG3’	57	32
RX1	F5’CCCCAACAGGAGCATTTAGAAGAC3’ R5’AGGGCACTCATGGCAGAAGGTT3’	60	30
HoxB9	F5’ TACTTACGGGCTTGGCTGGA3’ R5’ AGCGTGTAACCAGTTGGCTG3’	68	26
23,480	F-5’-ACAGGAGAAGGCATCAGACATGGAAC-3’ R-5’-GGATGCAATATCCTTTGGGATTCATCT-3’	61	28
16,875	F-5’-AATGTCTCAAGGCAGAGG-3’ R-5’-GTGTCACTGACACCAGAA-3’	46	28

### Reporter constructs

Reporter constructs including triple-repeated BMP4-response element (BRE) in pGL-2 basic plasmid (Kumar et al. 2018), activin-response element (ARE) and stem-cell like (SCL) in pGL-3 basic plasmid (Lee et al. 2012) were used for the reporter assays.

### Sample preparation and microarray analysis

Embryos after fertilization were grown until stage 8. Animal caps were dissected at stage 8-9, treated with activin (25 ng/ml) and cultured to stage 11 in 67% Leibovitz L-15 medium (GIBCO/BRL, Carlsbad, CA) with L-glutamine (0.3 mg/ml), 7 mM Tris-HCl (pH 7.5) and gentamicin (50 g/ml). About 500 animal caps were harvested and stored in the RNA*later*, an RNA stabilization reagent (Qiagen, Germantown, MD) at 4°C until RNA extraction. Total RNA was extracted from the animal caps with RNAse Mini kit (Qiagen) following the manufacturer’s instructions. Microarray experiments were performed by Seoulin Bioscience (Seongnam, Korea) with Affymetrix *Xenopus* Genome Gene Chip (Santa Clara, CA).

### RNA isolation and reverse transcription polymerase chain reaction (RT PCR)

Either of the mRNA for *smad2* (1 ng/embryo), *fgf8a* mRNA (1 ng/embryo), *fgf8b* (1 ng/embryo), DNFR (1 ng/embryo), or *dusp1* (3 ng/embryo) was injected into the animal pole at the one or 2-cell stage of *Xenopus* embryos and cultured in 30% MMR solution. Animal caps were then dissected from the injected and uninjected embryos and incubated until stage 11 and 24 in 1X L-15 growth medium. Total RNA was isolated from whole embryos and AC using RNA-Bee reagent following the manufacturer’s instructions (Tel-Test, Friendswood, TX) and it was treated with DNase I to remove genomic DNA contamination. RT–PCR was performed with Superscript II (Invitrogen, Carlsbad, CA) as recommended by the manufacturer using 2 μg of total RNA per reaction. PCR was performed according to the following conditions: 30 s at 94°C, 30 s at each annealing temperature, 30 s at 72°C, and 20–30 cycles of amplification. EF-1α and/or ODC was used as control to normalize the amount of cDNA used.

### Western blotting

The mRNAs were injected at the one-cell stage of embryos and collected at stage 11 for western blots. Non-injected embryos served as the negative control. Collected embryos were lysed in lysis buffer with phosphatase and protease inhibitors, in preparation for resolving the proteins with 10% SDS-PAGE and transfer to a PVDF membrane. The PVDF membranes post transfer were first blocked and then incubated with either pJnk, pErk, pan-Erk, pSmad1C (CS-9511S), pSmad1L (CS-9553P) and pSmad2C antibodies (Cell Signaling, Danvers, MA). Following washes of the PVDF membrane, it was incubated with the enzyme-labeled secondary antibody (ADI-SAB-300, Enzo Biochem, Farmingdale, NY). The protein signals were visualized by an ECL detection kit (GE Healthcare, Chicago, IL).

### Luciferase assays

Relative promoter activities were measured using a luciferase assay system according to the manufacturer’s instructions (Promega, Madison, WI). Five different groups of embryos (3 embryos per group) were harvested and homogenized in 10 μl lysis buffer per embryo. Embryo homogenates at 10 μl each were assayed with 40 μl luciferase substrate and the reporter gene activity was read by an illuminometer (Berthold Technologies, Bad Wildbad, Germany). All experiments were repeated at least three times for independently derived sample sets.

### Statistical analysis

Data were analyzed by GraphPad Prism5 (GraphPad, San Diego, CA). Statistical analysis was via one-way ANOVA with *p*< 0.05 instances considered to be significant differences. Notations: **, *p* ≤ 0.01; ***, *p* ≤ 0.001; ns, not significant.

## Results

### Activin/Smad2 induces fgfs and Fgf signal inhibitor dusp1 in AC

**Figure 1.**
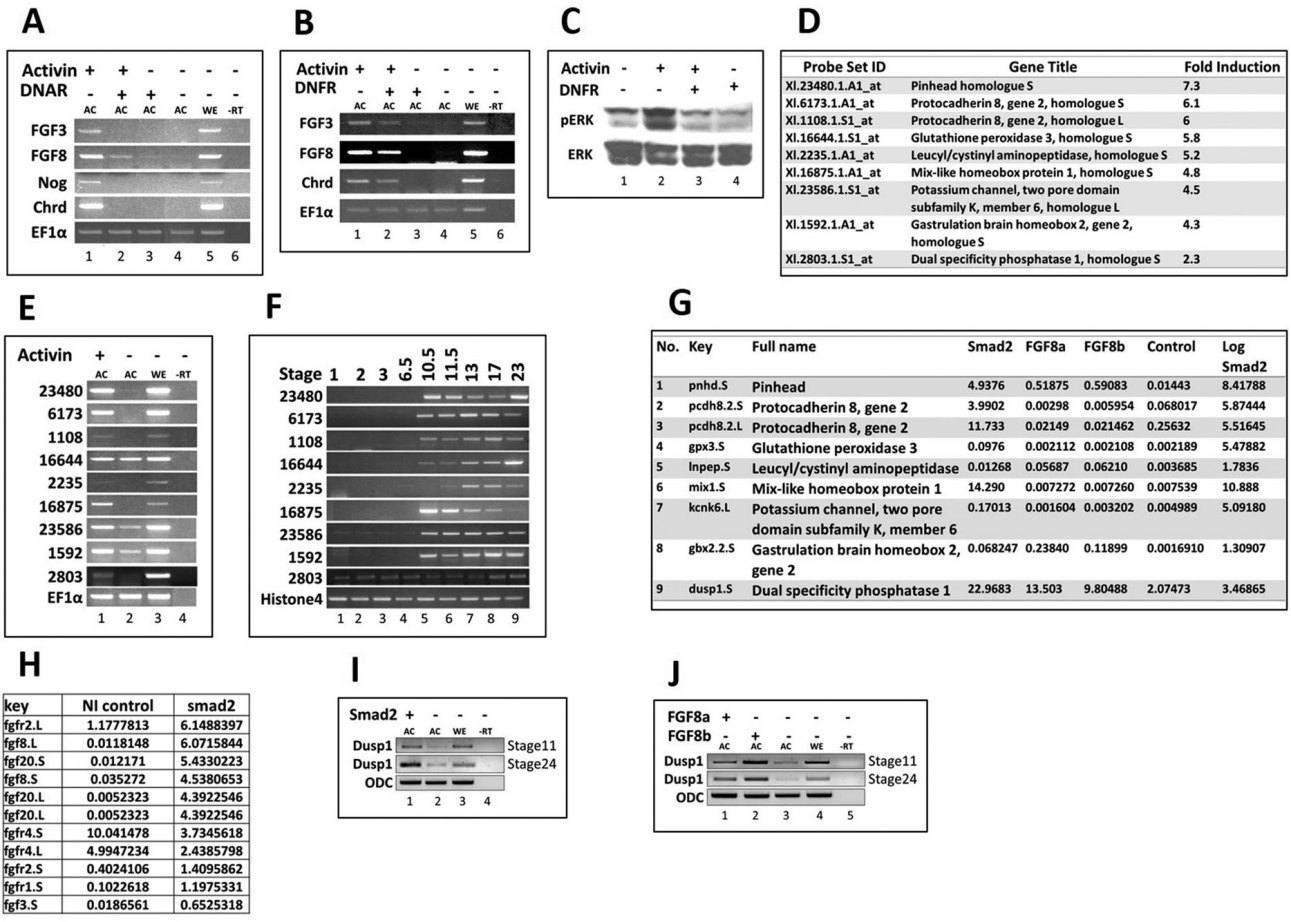
Ectopic expression of Activin, Smad2, and Fgfs in AC. All mRNAs (1 ng/embryo) were injected at the one-cell stage. The animal-caps were dissected at stage 8 and experiments were performed at stage 11 of *Xenopus* embryos. (A–C) DNAR and DNFR were injected separately and animal caps (AC) were dissected. ACs were treated with activin (25 ng/ml). The relative gene expressions were analyzed by RT-PCR and immunoprecipitation for pan Erk and pErk. (D–E) The AC were treated with activin. The specific gene expressions were analyzed by Microarray Affymetrix *Xenopus* Genome Gene Chip and RT-PCR of selected ESTs. (F) Stage-dependent spatial/ temporal expression of selected ESTs in whole embryos. (G–H) Fgf8a, Fgf8b and Smad2 were injected separately for RNA-Seq analysis and RT-PCR in AC.

Activin roles in mesoderm and neural development have been previously elaborated (Dyson and Gurdon 1997; Rodriguez-Martinez and Velasco 2012). However, the various roles of activin in development remain to be fully explored. To study the roles of activin/Smad2 pathway in germ layer specification of *Xenopus* embryos, we examined gene profiles by RT–PCR in activin (25 ng/ml) treated AC (stage 11). The increased expression of *chordin* and *noggin* was confirmed as positive control in activin treated AC samples (Figure 1(a); 3rd and 4th lines). The elevated expressions of *fgf3* and *fgf8* were also observed (Figure 1(a); 1st and 2nd lines). To define a role for these Fgf signals in activin induced gene expression, we injected DNAR (inhibitor of activin signaling) or DNFR (inhibitor of Fgf signaling) to *Xenopus* embryos at the one-cell stage and the ACs were dissected at stage 8. The explants were treated with activin (25 ng/ml) and grown until stage 11. In RT–PCR analysis, injection of either DNAR (Figure 1(a)) or DNFR (Figure 1(b)) reduced the expression of *fgf3*, *fgf8* and *chordin* (Figure 1(a and b); 2nd lanes). To examine whether activin activates the Fgf/Erk pathway, phosphorylation of Mapk was examined. Phosphorylated Mapk protein levels were increased in activin treated AC and decreased in presence of DNFR (Figure 1(c); 2nd and 3rd lanes). To identify additional genes involved in activin/Smad2 induced germ layer specification, we adapted two separate approaches of Affymetrix gene chip profiling for activin treated and transcriptome analysis for *smad2* treated AC. The Affymetrix gene chip gene expression profiling analyzed 14,400 gene transcript changes for activin (25 ng/ml) treated AC (stage 11). Several newly induced genes were identified (Figure 1(d)) with the increased gene expression in activin treated AC. The stage dependent expression patterns of the candidate genes are shown in Figure 1(d) as they were also confirmed by RT–PCR (Figure 1(e and f)). One of the induced genes in the activin treated gene chip set, namely Xl. 2803.1.S1, was identified as *dusp1* (Figure 1(d)). RT–PCR analysis at different developmental stage of *Xenopus* embryos confirmed that the temporal expression of *dusp1* started at the one-cell stage (maternal expression) and was sustained until the tailbud stage (stage 24) (Figure 1(f)). We next examined AC transcriptome post *smad2*, *fgf8a* and *fgf8b* mRNA injections at stage 11. *Dusp1* expression was increased in *smad2*, *fgf8a* and *fgf8b* samples with transcriptome analysis (Figure 1(g)). In addition, expression of *fgf* family members including *fgf2*, *3*, *8* and *20* were increased in *smad2* transcriptome analysis (Figure 1(h)). Transcripts of both isoforms of *fgf8. fgf8L* and *fgf8*S, were increased in *smad2* treated samples (Figure 1(h); lane 2nd and 4th). We confirmed the induction of *dusp1* in *smad2*, *fgf8a* and *fgf8b* injected AC using RT–PCR (Figure 1(i and j)). These findings suggested that activin/*smad2* promoted Fgf/Mapk phosphorylation via increased expression of *fgf*s. At the same time, activin/*smad2* and *fgf8a*/*b* increased expression of *dusp1*, an inhibitor gene of Fgf/Mapk pathway, indicating an intricate modulation of activin and Fgf signaling during activin/Smad2 induced germ layer specification of dorsal mesoderm, endoderm and neural tissue.

### Ectopic expression of dusp1 mimics DNFR in injected embryos

**Figure 2.**
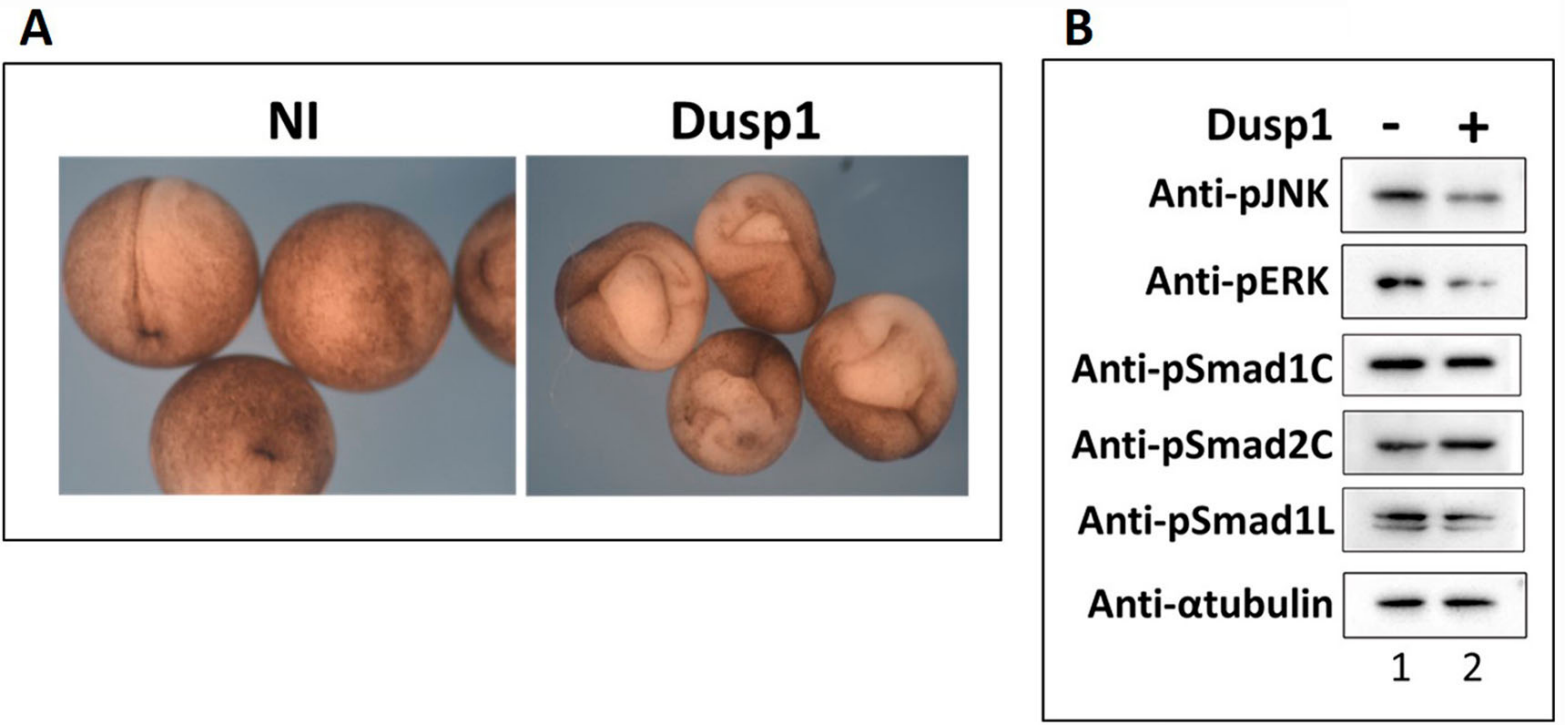
Overexpression of *dusp1* mimics the DNFR function. (A) *Dusp1* mRNA (3 ng/embryo) was injected at the 4 cell stage in dorsal half and harvested at stage 15 to identify the morphological change as compared to that of control (uninjected whole embryos). (B) *Dusp1* (3 ng/embryo) was injected and harvested at stage 11-11.5. Immunoprecipitation was performed with pJnk, pErk, pSmad1C (c-terminal), pSmad1L (Linker region), pSmad2C (c-terminal) and α-tubulin was included as a control (α-tubulin being a housekeeping protein).

Dual-specificity phosphatase (Dusp) family proteins are so named for their ability to dephosphorylate both threonine/serine and tyrosine residues of their targets that include Mapk/Erk and Jnk (Keyse 2008; Huang and Tan 2012; Shen et al. 2016). To identify the role of *dusp1* during early embryogenesis, we injected *dusp1* mRNA in the dorsal side of *Xenopus* embryo at the 4-cell stage and a severe gastrulation defect was observed, mimicking *DNFR* injected embryos (Figure 2(a)). To see whether *dusp1* could affect Fgf mediated Mapk/Erk and Jnk activation in *Xenopus* embryos, we evaluated phosphorylated levels of Mapk/Erk and Jnk by western blotting in *dusp1* injected *Xenopus* embryos and noticed decreased levels of phosphorylated Mapk/Erk and Jnk in presence of increased *dusp1* expression (Figure 2(b); line 1st and 2nd). We also observed decreased level of Smad1 linker region phosphorylation with the phosphorylation known to be mediated by Mapk/Erk (Figure 2(b); line 5th). Collectively, the results indicate that *dusp1* negatively affects Fgf signaling during early *Xenopus* embryogenesis.

### Dusp1 inhibits Smad2 induced dorsal mesoderm in AC

**Figure 3.**
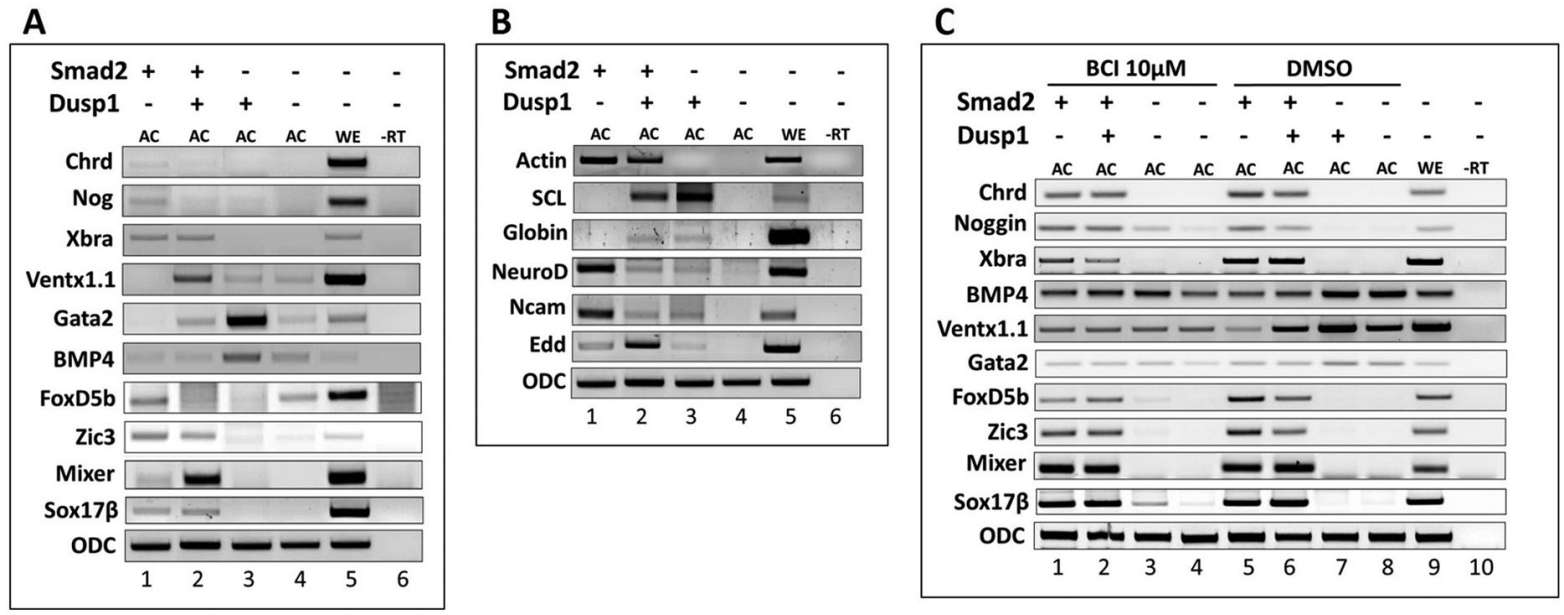
*Dusp1* inhibits Smad2 mediated dorsal mesoderm (organizer) in AC. (A–C) *Smad2* mRNA (1 ng/embryo) was injected separately or co-injected with *dusp1* (3 ng/embryo) at the one-cell stage, followed by dissection of the AC (AC) and harvested at stage 11-11.5. Relative gene expression was analyzed by RT-PCR. (C) BCI 10 µM (Dusp inhibitor) and DMSO as control, treated at stage 8.

To analyze the role of *dusp1* in activin/Smad2 mediated germ layer specification and whether *dusp1* stimulates activin/Smad2 activity of organizer and neural gene expression or inhibits them, we measured several neural and mesodermal markers in *smad2* and *dusp1* over-expressed conditions in AC at stage 11 and 24. Interestingly, we found that overexpression of *dusp1* inhibited *smad2* mediated expression of organizer genes including *chordin* and *noggin* as well as early and late neural markers such as *foxdl4 1.1*, *zic3*, *ncam* and *neuroD* (Figure 3(a and b)). *Dusp1* mRNA injection alone or along with *smad2* increased the expression of early ventral mesoderm markers including *bmp4*, *ventx1.1*, *gata2* at stage 11 and of later ventral mesoderm (blood island) genes including *globin* and *scl* at stage 24 (Figure 3(a and b)). In addition, *dusp1* co-injection with *smad2* increased early (*mixer* and *sox17β*) and later endoderm (*edd*) makers (Figure 3(a and b)).

To evaluate a role for *dusp1* loss in the animal cap explant system, *smad2* alone or *smad2* with *dusp1* were injected at the one-cell stage. The ACs were dissected at stage 8 and treated with BCI (a known inhibitor of Dusp1) or DMSO (as negative control). The explants were grown until stage 11 and subjected to RT–PCR analysis. BCI blocked the altered expression of early neural markers including *zic3* and *foxD4l1.1*, along with dorsal mesoderm markers including *chordin* and *noggin*, ventral mesoderm markers including *ventx1.1*, *gata2* and *bmp4,* and endoderm markers including *mixer* and *sox17β* (Figure 3(c)). This indicated that *dusp1* had specific roles in *smad2* induced early germ layer specification. Taken together, we found *dusp1* as an inhibitor of activin/*smad2* induced dorsal and neural tissue formation and as a promoter of ventral mesoderm and endoderm fate together with *smad2*.

### Dusp1 inhibits fgf8 induced neural induction and modulates anterior-posterior patterning for neural tissue formation

**Figure 4.**
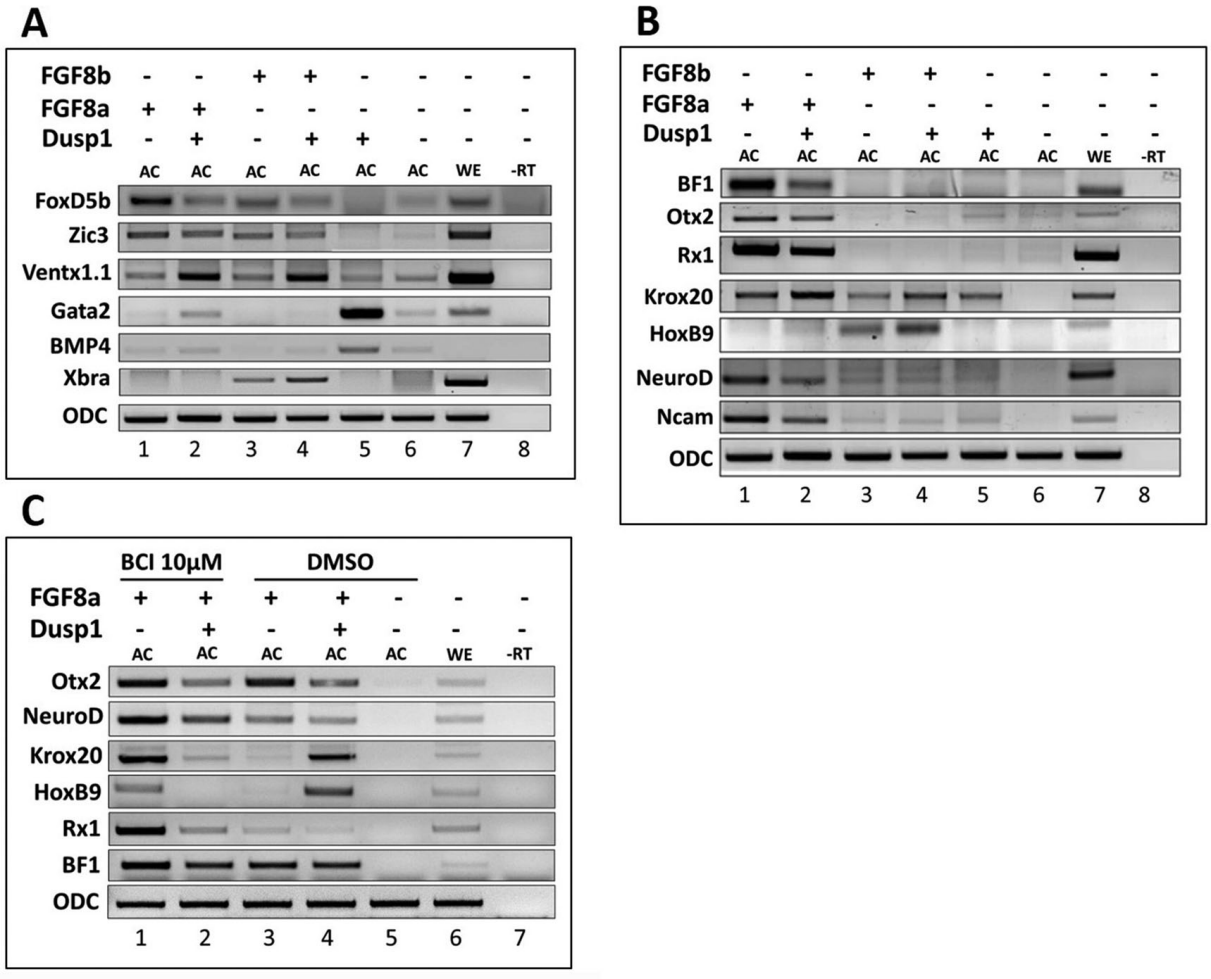
*Dusp1* inhibits Fgf8 induced neural induction and modulates anterior-posterior patterning of neural tissue formation. (A–C) *Fgf8a* and *fgf8b* mRNA (1 ng/embryo) were injected separately or co-injected with *dusp1* (3 ng/embryo) at the one-cell stage, followed by dissection of the AC (AC) at stage 8 and harvested at stage 11 and 24. RT-PCR was performed to examine the expression of target genes to compare with the control (uninjected AC), (C) BCI (Dusp1 inhibitor, 10 µM and DMSO as control) treatment at stage 8.

*Dusp1* inhibited the Smad2 induced organizer gene and neural gene expression (Figure 3(a)). We next examined whether the presence of *dusp1* influenced *fgf8a* and *fgf8b* mediated gene expression changes. In RT–PCR analysis, *fgf8a*/*b* increased expression of early neural genes including *foxd4l1.1* and *zic3* (Figure 4(a))*. Foxd4l1.1* expression was decreased by co-injection with *dusp1*. On the other hand, *zic3* (another early neural gene) was not as much affected compared with *foxd4l1.1* (Figure 4(a))*.* Expression of a neural repressor gene *ventx1.1* was increased by co-injection with *fgf8a/b* and *dusp1* when compared to *ventx1.1* expression with *fgf8a/b* alone or uninjected AC (Figure 4(a)). At stage 24, *dusp1* downregulated *fgf8a* mediated anterior neural genes including *bf1*, *otx2* and *rx1* accompanied with a reduction in *neurod* and *ncam* expression. However, *fgf8b* or *fgf8b* when in presence of *dusp1* did not lead to induction of the anterior neural genes. In contrast to *fgf8a*, *fgf8b* injection did not induce the neural genes including *bf1*, *otx2* and *rx1* and general neural makers including *neurod* and *ncam* (Figure 4(b)). Interestingly, *dusp1* upregulated expression of midbrain maker gene *krox20* and posterior neural maker gene *hoxb9* in conjugation with *fgf8a/b* (Figure 4(b)).

For a possible role for *dusp1* in anterior-posterior neural patterning, gene expression changes at a late stage (stage 24) in presence and absence of BCI were examined. Increased expression of *krox20* and *hoxB9* by co-injection with *fgf8a* and *dusp1* was reversed to the basal level as in *fgf8a* injected AC, indicating that BCI effectively blocked *dusp1* mediated caudualization activity (Figure 4(c)). Moreover, *krox20* and *hoxB9* expression was increased in BCI treated cases when compared to that without BCI treatment in *fgf8a* injected samples (Figure 4(c)). Taken together, these findings indicate that *dusps* may be a modulator of Fgf8 signaling, balancing the extent of anterior to posterior neural patterning.

### Dusp1 modulates reporter activities of both activin and bmp response elements

**Figure 5.**
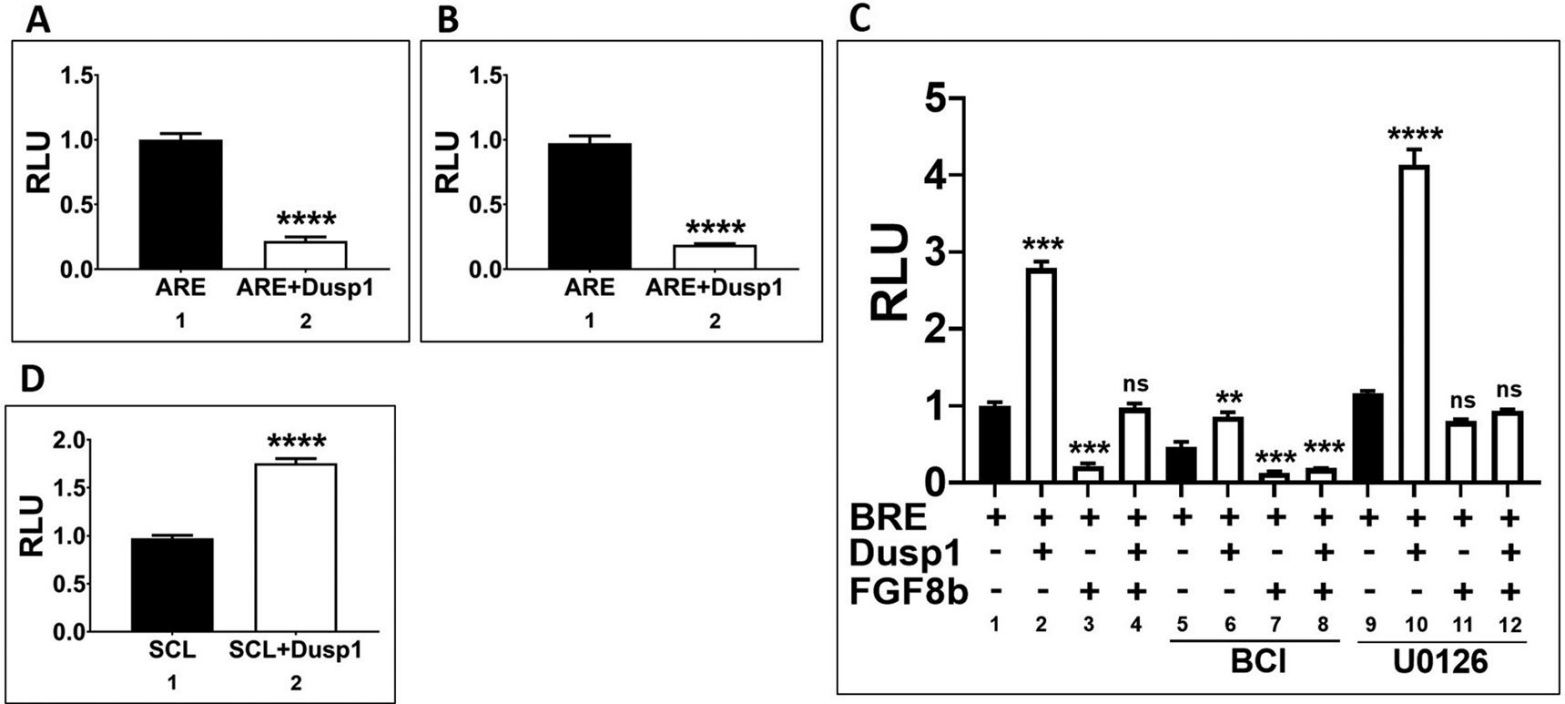
*Dusp1* regulates reporter activities of ARE, BRE and SCL promoter constructs. (A and B) The reporter ARE construct (40 pg/embryo) was injected with or without *dusp1* (3 ng/embryo) at the one-cell stage and the embryos were grown until stage 11 and 18 for the relative reporter activity. (C) The reporter BRE construct (40 pg/embryo) was injected with or without *dusp1* (3 ng/embryo) and/or *fgf8b* (1 ng/embryo) or together (with or without treatment with BCI and U0126) and the embryos at stage 11 to measure the relative reporter activity. (D) SCL promoter (40 pg/embryo) was injected with or without *dusp1* (3 ng/embryo) at stage 11 to measure the relative promoter activity. The data are shown as mean ± S.E. of the values from at least three independent experiments. Differences were considered significant at *P* < 0.05.

*Dusp1* inhibited activin/*smad2* mediated organizer gene expression and *fgf8* mediated neural induction. As *dusp1* decreased activin and *fgf8* induced germ layer genes for organizer and neural specification, we next assayed activin specific response element (ARE) reporter luciferase activity in presence of *dusp1*. A reporter of 3x repeated ARE (40 pg/5 nl) was injected with or without *dusp1* (3 ng) at the one-cell stage. The embryos were grown until stage 11 (Figure 5(a)) and 18 (Figure 5(b)) to measure their relative reporter activities. *Dusp1* injection decreased the relative reporter activity of ARE up to 6-fold at both stage 11 and 18 as compared to control (Figure 5(a and b)). We then examined the reporter activity for BRE. A reporter construct of 3x repeated BRE (40 pg/5 nl) was injected with or without *dusp1* (3 ng) at the one or 2 cell stage. The reporter (40 pg/5 nl) was also injected under various conditions including with or without *dusp1* (3 ng/5 nl) or *fgf8*b (1 ng/5 nl) or together at the one-cell stage, and treated with or without BCI and U0126 at stage 8 until harvesting of the embryos at stage 11. As expected, *dusp1* upregulated the activity of BRE (Figure 5(c); 1st and 2nd bar). On the other hand, *fgf8b* decreased BRE activity (Figure 5(c); 1st and 3rd bar); however, this decrease returned to basal levels by *dusp1* co-injection (Figure 5(c); 1st, 3rd and 4th bar), implying that the block of changes was related to the kinase activity of Dusp1 on Fgf8b signaling. To evaluate the involvement of Dusp1 kinase activity on the BRE reporter, the similar samples were co-treated with BCI (a Dusp1 inhibitor) (Figure 5(c); 5th–8rd bars). In those, BRE reporter activity decreased by BCI treatment alone, indicating that endogenous Dusp1 is a positive regulator of BRE activity (Figure 5(c); 2nd bar). In addition, the inhibitory effect of Fgf8b/Erk on BRE reporter activity was blocked by BCI, indicating that BCI effectively blocked Dusp1 activity (Figure 5(c); 7th and 8th bar). Inhibition of Fgf8b intracellular signaling by the Erk inhibitor U0126 was also evaluated in the context of BRE reporter activity changes. In presence of U0126, BRE reporter activity was higher with *dusp1* co-injection, indicating that co-inhibition of Erk activity by Dusp1 and U0126 further increases BRE (Bmp4/Smad1 response element) reporter activity. In addition, Fgf8b mediated inhibition of BRE activity was blocked to that of control levels in presence of U0126 (Figure 5(c), 3rd and 11th bar). We concluded that BRE reporter activity was respectively stimulated by *dusp1* or inhibited by *fgf8b,* blockable to basal levels by BCI or U0126, respectively. *Dusp1* also increased the expression of *scl* (Figure 3(b)), and we next examined effect *of dusp1* expression on the reporter activity for the *scl* promoter. The relative reporter activity of *scl* promoter construct increased by *dusp1* similar to that of its increased expression (Figure 3(b), 3rd lane of 2nd line). Taken together, *Dusp1* negatively regulated ARE and positively regulated BRE reporter activity.

## Discussion

In the present study, we attempted to define specific genes regulated in activin/*smad2* mediated germ layer specification. We identified *dusp1*, known as a negative modulator of Fgf signaling in mammals. We examined the role of *dusp1* in early *Xenopus* embryos, having three questions in mind. First, whether *dusp1* was a positive or a negative modulator for Fgf signaling in *Xenopus* embryos. Second, whether *dusp1* modulated activin/*smad2* and *fgf8* mediated germ layer specification, and third, whether *dusp1* also modulated Bmp4/Smad1 signaling. We found that Dusp1 was a negative modulator of Fgf signaling; it negatively modulated activin/Smad2 signaling and positively modulated that of Bmp4/Smad1. However, these findings were somewhat unexpected as activin/Smad2 usually increases dorsal mesoderm and neural genes for which it is necessary. As such, we suggest that Dusp1 is a finetuning modulator of Fgf/Erk signaling, making Dusp1 a necessary molecule in regulating germ layer specification by Fgf/Mapk, activin/Smad2 and Bmp/Smad1 signaling, providing a context dependent outcome in complex molecular environments. The implication and interpretation of this study is discussed below in terms of the crosstalk among the above three signaling pathways for germ layer specification in *Xenopus* embryos.

### Activin/smad2 signaling leads to dorsal mesoderm and neural formation with modulation of fgf signaling

Our present study provided substantial evidence in support the hypothesis that activin/Smad2 play a key role in regulation of Fgf signaling outcome. In this study we found that activin/*smad2* increased the expression of various Fgfs including *fgf2*, *3*, *8* and *20*, shown in *smad2* transcriptome analysis (Figure 1(h)), shown with the expression of *fgf3* and *fgf8* specifically blocked by DNAR (Figure 1(a)). In addition, the expressions of *fgf3* and *fgf8* were reduced by DNFR injection (Figure 1(a and b); 2nd lanes). Activin/Smad2 treatment usually increases organizer genes including *chordin* and *noggin*. For this dorsal tissue specification, the requirement Fgf signaling has been previously addressed (Kimelman and Kirschner 1987; Cornell and Kimelman 1994), in addition to the finding that Fgf inhibition leads to repression of organizer gene expression (Kimelman and Kirschner 1987; Cornell and Kimelman 1994). In the present study, we provide additional evidence of an interrelationship between activin/Smad2 and Fgf signaling pathways as activin/Smad2 signaling induced expression of Fgfs including Fgf2,3,8 and 20. We postulate that activin/*smad2* requires an initial Fgf signal for organizer gene expression and then it newly induces each Fgf, further contributing to different germ layer specification for dorsal mesoderm, neuroectoderm and endoderm formation. The postulate of the initial requirement of the Fgf signal was supported with our result that early injection of DNFR abolished organizer (*chordin*) and Fgf expression (*fgf3* and *fgf8*) (Figure 1(a)). Presently, however, the specific role of individual Fgf’s in different germ layer specification remains undefined and awaits more studies.

### Activin/Smad2 induces dusp1 and dusp1 finetunes outcome for Fgf as well as activin/smad2

From both Affimetrix gene chip and transcriptome analysis of activin/*smad2* treated AC, *dusp1* was consistently upregulated (Figure 1(d and g)). In addition, Fgf8 also increased *dusp1* (Figure 1(j)) which inhibited phosphorylation of Jnk, Erk and Smad1 linker region (Figure 2(b)) in embryos. The expression of *dusp1* was unexpected since activin/Smad2 stimulated Fgf signaling with increased Fgf/Erk phosphorylation (Figure 1(c)) which was dependent on an Fgf signal. Dependence on an Fgf signal for Erk phosphorylation was confirmed by co-injection with DNFR. DNFR reduced Erk phosphorylation (Figure 1(c); 3rd lane). We also confirmed the Fgf signal inhibition activity of *dusp1* by showing a gastrulation defect in whole embryos (Figure 2(a)), a phenotype similar to that of DNFR injected embryos. *Dusp1* induction by activin/Smad2 and Fgf8 led us to postulate that Dusp1 might be possibly finetuning activin/Smad2 and Fgf8 induced specific germ layer formation in terms of directly affecting the signaling molecules including Jnk, Erk and Smad1 (Figure 2(b)). Therefore, we evaluated *dusp1* effects on induced germ layer specification by Smad2 (Figure 3) and Fgf8 (Figure 4).

Cooperative input of Fgf with Tgf-ß induces a mesoderm specific transcript *muscle actin* in animal cap explant of *Xenopus* embryos, and previously, there has been one instance of Fgf signaling being required for normal dorsal mesoderm formation by activin/Smad2 (Cornell and Kimelman 1994). In our previous study, inhibition of Fgf signaling converted activin/Smad2 induced dorsal mesoderm to the ventral mesoderm (Lee et al. 2011). Thus, as we expected, *dusp1*, an inhibitor of Fgf signaling, retained the converting activity of dorsal to ventral mesoderm with Smad2 (Figure 3(a); compare 1st and 2nd lane). In addition, we found that *dusp1* alone displayed a strong blood formation (ventral mesoderm) activity including induction of early (*gata2* and *Bmp4*) and late ventral genes (*scl* and *globin*) (Figure 3(a and b)) in AC. Furthermore, *dusp1* and *smad2* co-injection significantly increased early (*mixer* and *sox17β*) (Figure 3(a)) and late endoderm (*edd*) (Figure 3(b)) genes, suggesting that *dusp1* modulates activin/Smad2 mediated germ layer specification. We postulate that *dusp1* induced by activin/Smad2 may have a role in modifying the signal for facilitating one of the germ layers, particularly for endoderm formation. One similar discovery has recently been reported where the related *dusp4* was necessary for the endoderm program in *Zebrafish* embryos (Brown et al. 2008). We also evaluated *dusp1* effect on Fgf8a and Fgf8b induced gene expression (Figure 4) and found that *dusp1* inhibited neuroectoderm formation with upregulation of the neural repressor gene *ventx1*.1 and modulation of anterior-posterior patterning genes of neural differentiation (Figure 4(b)). We were interested in evaluating *dusp1* effect on Fgf8 induced anterior-posterior neural genes since Fgf signaling has been proposed as the caudalization signal for neural patterning. However, *dusp1*, being an inhibitor of Fgf signaling, unexpectedly did not lead to an increase of anterior neural genes including *bf1*, *otx2* and *rx1* (Figure 4(b)). *Dusp1* actually increased posterior neural genes including *krox20* and *hoxb9* for an unknown reason (Figure 4(c); 4th lane). We previously postulated that anterior neural formation may not be the default pathway and BMP inhibition may be accompanied with expression of antagonizers of posteriorizing factors including retinoic acid, Fgf and wnt. Dusp1 was one of antagonizing molecules of Fgf posteriorization modulator. However, Dusp1 was not the expected molecule involved in anteriorlizing pathway elicited by BMP inhibition and Fgf8a. Interestingly, both Dusp1 and BCI, treatment lead to posteriorization of neural tissues with increases in *krox20* and *hoxb9*, indicating that other *Dusp*s as nonspecific targets of BCI may be the genes involved in anterior patterning of neural differentiation. Overall, Dusp1 or its homologs may have roles in endoderm formation for activin/Smad2 signaling and in anterior-posterior patterning for that of DNBR/Fgf8.

### Dusp1 modulates both activin/smad2 and Bmp/Smad1 pathways

We asked whether as an inhibitory modulator of Fgf signaling, Dusp1 regulates both a dorsal signal pathway activin/Smad2 and a ventral signal Bmp4/Smad1. We used a direct response cis-acting element of Smad2 (ARE) and Smad1 binding (BRE) (Kumar et al. 2018) respectively to examine the *dusp1* effect. We expected a decrease in ARE activity and an increase in BRE activity by *dusp1* based on the results of inhibition of organizer gene expression and Smad1 linker region phosphorylation by *dusp1,* respectively. As expected, *dusp1* decreased the reporter activity of ARE and increased that of BRE (Figure 4(a–c)), thus supporting our hypothesis that *dusp1* acts as a finetuning modulator in a context dependent manner. Moreover, previously shown, an increase in the relative luciferase activity of stem cell like (Scl) promoter (Lee et al. 2012) indicates that *dusp1* induces ventral mesoderm formation. BCI, an inhibitor of Dusps (Ramkissoon et al. 2019), also decreased BRE reporter activity according to our data, indicating that endogenous *dusp1* may have a role in modulating Bmp/Smad1 signaling.

**Figure 6.**
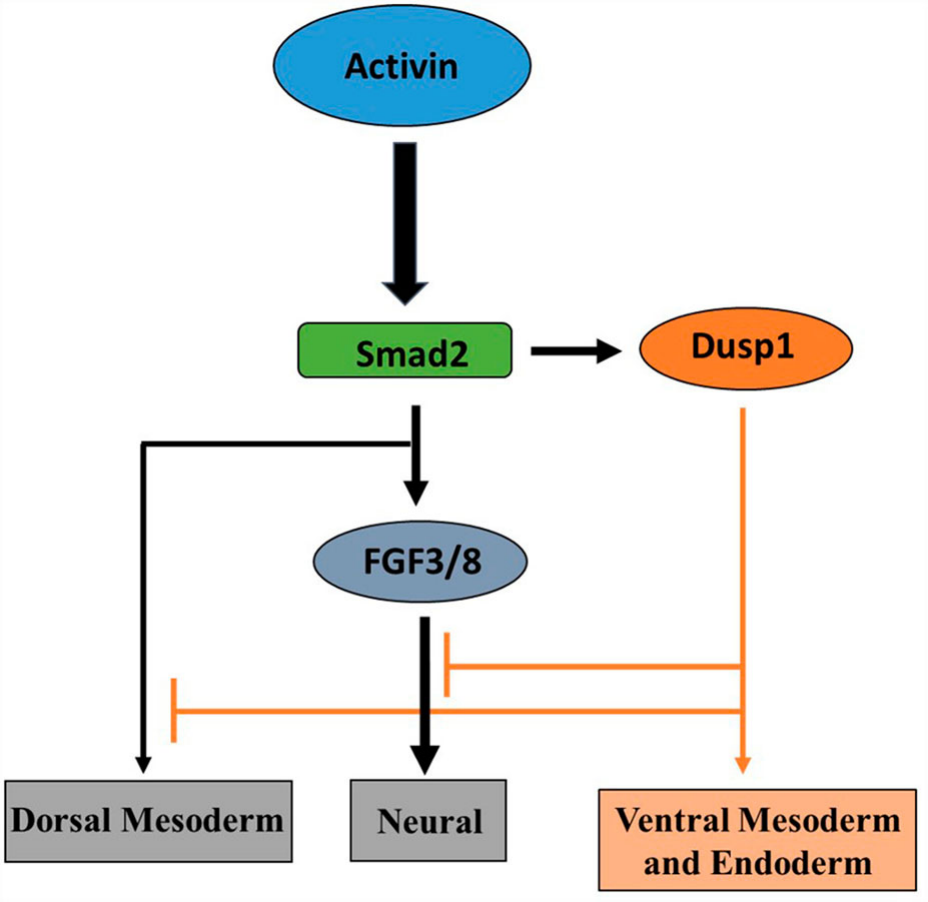
Schematic model: *Dusp1* converts activin/Smad2 mediated neuro-ectoderm and dorsal mesoderm to ventral mesoderm and endoderm in AC of *Xenopus* embryos.

In summary, we propose a model of *dusp1* modulating Fgf, activin/Smad2 and Bmp4/Smad1 signaling in germ-layer specification (Figure 6). In this paper, we provided evidence of *dusp1* acting as a finetuning modulator in dorso-ventral and anterior-posterior neutral patterning during the germ-layer specification of *Xenopus* embryos. Taken together, our finding recognizes Dusp1 as a regulatory factor in activin, Bmp and Fgf combinatorial signaling in early embryogenesis. This study provides a better understanding of the signaling networks for the cell under various physiological conditions.

## References

[CIT0001] Asashima M, Nakano H, Shimada K, Kinoshita K, Ishii K, Shibai H, Ueno N. 1990. Mesodermal induction in early amphibian embryos by activin A (erythroid differentiation factor). Rouxs Arch Dev Biol. 198:330–335. Epub 1990/03/01. 28305412 10.1007/BF00383771

[CIT0002] Brown JL, Snir M, Noushmehr H, Kirby M, Hong SK, Elkahloun AG, Feldman B. 2008. Transcriptional profiling of endogenous germ layer precursor cells identifies dusp4 as an essential gene in zebrafish endoderm specification. Proc Natl Acad Sci U S A. 105:12337–12342. Epub 2008/08/23. 18719100 10.1073/pnas.0805589105 PMC2527912

[CIT0003] Christen B, Slack JM. 1997. Fgf-8 is associated with anteroposterior patterning and limb regeneration in Xenopus. Dev Biol. 192:455–466. Epub 1998/01/27. 9441681 10.1006/dbio.1997.8732

[CIT0004] Cornell RA, Kimelman D. 1994. Activin-mediated mesoderm induction requires Fgf. Development. 120:453–462. Epub 1994/02/01. 8149920 10.1242/dev.120.2.453

[CIT0005] Dyson S, Gurdon JB. 1997. Activin signalling has a necessary function in Xenopus early development. Curr Biol. 7:81–84. Epub 1997/01/01. 8999997 10.1016/s0960-9822(06)00030-3

[CIT0006] Feng XH, Derynck R. 2005. Specificity and versatility in tgf-beta signaling through Smads. Annu Rev Cell Dev Biol. 21:659–693. Epub 2005/10/11. 16212511 10.1146/annurev.cellbio.21.022404.142018

[CIT0007] Gurdon JB, Mitchell A, Mahony D. 1995. Direct and continuous assessment by cells of their position in a morphogen gradient. Nature. 376:520–521. Epub 1995/08/10. 7637784 10.1038/376520a0

[CIT0008] Heldin CH, Moustakas A. 2012. Role of Smads in TGFbeta signaling. Cell Tissue Res. 347:21–36. Epub 2011/06/07. 21643690 10.1007/s00441-011-1190-x

[CIT0009] Holm TM, Habashi JP, Doyle JJ, Bedja D, Chen Y, van Erp C, Lindsay ME, Kim D, Schoenhoff F, Cohn RD, et al. 2011. Noncanonical TGFbeta signaling contributes to aortic aneurysm progression in Marfan syndrome mice. Science. 332:358–361. Epub 2011/04/16. 21493862 10.1126/science.1192149 PMC3111087

[CIT0010] Huang CY, Tan TH. 2012. DUSPs, to MAP kinases and beyond. Cell & Bioscience. 2:1–10. Epub 2012/07/10. 22769588 10.1186/2045-3701-2-24 PMC3406950

[CIT0011] Isaacs HV, Pownall ME, Slack JM. 1994. Efgf regulates Xbra expression during Xenopus gastrulation. EMBO J 13:4469–4481. Epub 1994/10/03. 7925289 10.1002/j.1460-2075.1994.tb06769.x PMC395379

[CIT0012] Keyse SM. 2008. Dual-specificity MAP kinase phosphatases (MKPs) and cancer. Cancer Metastasis Rev. 27:253–261. Epub 2008/03/12. 18330678 10.1007/s10555-008-9123-1

[CIT0013] Kimelman D, Kirschner M. 1987. Synergistic induction of mesoderm by Fgf and TGF-beta and the identification of an mRNA coding for Fgf in the early Xenopus embryo. Cell. 51:869–877. Epub 1987/12/04. 3479265 10.1016/0092-8674(87)90110-3

[CIT0014] Kiyono K, Suzuki HI, Matsuyama H, Morishita Y, Komuro A, Kano MR, Sugimoto K, Miyazono K. 2009. Autophagy is activated by TGF-beta and potentiates TGF-beta-mediated growth inhibition in human hepatocellular carcinoma cells. Cancer Res. 69:8844–8852. Epub 2009/11/12. 19903843 10.1158/0008-5472.CAN-08-4401

[CIT0015] Kroll KL, Amaya E. 1996. Transgenic Xenopus embryos from sperm nuclear transplantations reveal Fgf signaling requirements during gastrulation. Development. 122:3173–3183. Epub 1996/10/01. 8898230 10.1242/dev.122.10.3173

[CIT0016] Kumar S, Umair Z, Yoon J, Lee U, Kim SC, Park JB, Lee JY, Kim J. 2018. Xbra and Smad-1 cooperate to activate the transcription of neural repressor ventx1.1 in Xenopus embryos. Sci Rep. 8:1–11. Epub 2018/08/01. 30061699 10.1038/s41598-018-29740-9 PMC6065435

[CIT0017] Lee SY, Lim SK, Cha SW, Yoon J, Lee SH, Lee HS, Park JB, Lee JY, Kim SC, Kim J. 2011. Inhibition of Fgf signaling converts dorsal mesoderm to ventral mesoderm in early Xenopus embryos. Differentiation. 82:99–107. Epub 2011/06/21. 21684060 10.1016/j.diff.2011.05.009

[CIT0018] Lee SY, Yoon J, Lee MH, Jung SK, Kim DJ, Bode AM, Kim J, Dong Z. 2012. The role of heterodimeric AP-1 protein comprised of JunD and c-Fos proteins in hematopoiesis. J Biol Chem. 287:31342–31348. Epub 2012/07/24. 22822070 10.1074/jbc.M112.387266 PMC3438963

[CIT0019] Luo K. 2017. Signaling Cross Talk between TGF-beta/smad and other signaling pathways. Cold Spring Harb Perspect Biol. 9:1–28. Epub 2016/11/12. 10.1101/cshperspect.a022137 PMC5204325 27836834

[CIT0020] Massague J. 2012. TGFbeta signalling in context. Nat Rev Mol Cell Biol. 13:616–630. Epub 2012/09/21. 22992590 10.1038/nrm3434 PMC4027049

[CIT0021] McDowell N, Zorn AM, Crease DJ, Gurdon JB. 1997. Activin has direct long-range signalling activity and can form a concentration gradient by diffusion. Curr Biol. 7:671–681. Epub 1997/09/01. 9285724 10.1016/s0960-9822(06)00294-6

[CIT0022] Piepenburg O, Grimmer D, Williams PH, Smith JC. 2004. Activin redux: specification of mesodermal pattern in Xenopus by graded concentrations of endogenous activin B. Development. 131:4977–4986. Epub 2004/09/17. 15371302 10.1242/dev.01323

[CIT0023] Ramkissoon A, Chaney KE, Milewski D, Williams KB, Williams RL, Choi K, Miller A, Kalin TV, Pressey JG, Szabo S, et al. 2019. Targeted inhibition of the dual specificity phosphatases DUSP1 and DUSP6 Suppress MPNST growth via JNK. Clin Cancer Res. 25:4117–4127. Epub 2019/04/03. 30936125 10.1158/1078-0432.CCR-18-3224 PMC6606396

[CIT0024] Rodriguez-Martinez G, Velasco I. 2012. Activin and TGF-beta effects on brain development and neural stem cells. CNS Neurol Disord Drug Targets. 11:844–855. Epub 2012/11/08. 23131163 10.2174/1871527311201070844

[CIT0025] Schulte-Merker S, Smith JC. 1995. Mesoderm formation in response to brachyury requires Fgf signaling. Curr Biol. 5:62–67. Epub 1995/01/01. 7535172 10.1016/s0960-9822(95)00017-0

[CIT0026] Shen J, Zhang Y, Yu H, Shen B, Liang Y, Jin R, Liu X, Shi L, Cai X. 2016. Role of DUSP1/MKP1 in tumorigenesis, tumor progression and therapy. Cancer Med. 5:2061–2068. Epub 2016/05/27. 27227569 10.1002/cam4.772 PMC4884638

[CIT0027] Slack JM, Darlington BG, Gillespie LL, Godsave SF, Isaacs HV, Paterno GD. 1990. Mesoderm induction by fibroblast growth factor in early Xenopus development. Philos Trans R Soc Lond B Biol Sci. 327:75–84. Epub 1990/03/12. 1969663 10.1098/rstb.1990.0044

[CIT0028] Smith JC, Price BM, Van Nimmen K, Huylebroeck D. 1990. Identification of a potent Xenopus mesoderm-inducing factor as a homologue of activin A. Nature. 345:729–731. Epub 1990/06/21. 2113615 10.1038/345729a0

[CIT0029] Xu X, Han J, Ito Y, Bringas P, Jr., Deng C, Chai Y. 2008. Ectodermal Smad4 and p38 MAPK are functionally redundant in mediating TGF-beta/BMP signaling during tooth and palate development. Dev Cell. 15:322–329. Epub 2008/08/13. 18694570 10.1016/j.devcel.2008.06.004 PMC2610417

